# A tolerability burden index in schizophrenia: incorporating patient perspective in clinical trial adverse event reporting

**DOI:** 10.1080/20016689.2017.1372026

**Published:** 2017-09-13

**Authors:** Clément François, Alice Guiraud-Diawara, Christophe Lançon, Pierre Michel Llorca, Ann Hartry, Lene Hammer-Helmich, Djamel A. Zighed, Adrian Tanasescu, Mondher Toumi

**Affiliations:** ^a^ Health Economics Outcome Research, Lundbeck, Deerfield, IL, USA; ^b^ Health Economics Outcome Research, Lundbeck SAS, Issy-les-Moulineaux, France; ^c^ Laboratoire de Santé Publique Évaluation des Systèmes de Soins et Santé Perçue, Université de la Méditerranée – EA 3279 – Faculté de Médecine, Marseille 5, France; ^d^ Centre Hospitalier Universitaire Clermont-Ferrand, EA 7280 Université Clermont-Auvergne, Clermont Ferrand, France; ^e^ Health Economics Outcome Research, Lundbeck A/S, Valby, Denmark; ^f^ ERIC Lab, Université de Lyon, Bron, France; ^g^ Rithme Consulting, Villeurbanne, France; ^h^ Department of Public Health, Aix Marseille University, Marseille 5, France

**Keywords:** Adverse event, antipsychotic, schizophrenia, standardization, tolerability index

## Abstract

**Background**: Adverse event (AE) reporting in clinical trials does not fully capture the patient-level perspective and comparing tolerability across treatments or among studies is difficult.

**Objective**: This study was designed to develop a treatment tolerability index algorithm that combines AE reporting with physician- and patient-level AE information into a global burden score to allow comparison of the overall tolerability of antipsychotic medications used in treating schizophrenia.

**Study design**: Data from a 4-arm, placebo-controlled clinical trial were used in the proposed tolerability index algorithm. For each patient, AEs were adjusted by frequency, severity, duration, and patient-experienced importance, and average tolerability-related burden scores were calculated.

**Setting**: Algorithm development analyses.

**Patients**: This study analyzed data from a previously completed clinical trial that evaluated a potential antipsychotic medication; no patients were involved in the current study.

**Intervention**: No interventions were administered in this study; the analyses described used data derived from a previously completed clinical trial in which patients received bifeprunox, risperidone, or placebo.

**Main outcome measure**: Burden scores and tolerability index scores were compared for patients who did or did not discontinue treatment because of AEs.

**Results**: The number of AEs varied widely among patients. Burden scores were significantly worse for patients who discontinued treatment because of AEs. Mean tolerability index scores, adjusted based on AE frequency, severity-adjusted duration, and patient-experienced impact, were poorer for active medications than placebo, and increased with dose.

**Conclusion**: The treatment tolerability index will allow comparison of AE burden and tolerability between treatments using existing clinical trial information. This suggests that scoring is possible, is clinically relevant, and allows standardized comparison of antipsychotic tolerability.

## Introduction

Reporting of adverse events (AEs) in randomized clinical trials is comprehensive, but the information reported is frequently limited to population-level summaries of AE frequency and severity. Current reporting methods are thorough and adequate for establishing product safety but may not fully reflect the impact of non-serious AEs on an individual patient level. Additionally, AE reports do not account for patient perceptions of the overall burden of treatment associated with AEs (single or multiple occurrences) and tolerability. Instead, information about patient experiences as reported by clinicians and aggregated for each AE is most commonly presented, without documenting the cumulated impact experienced by a single patient or the patients who do not experience any such events. This limitation of AE reporting in clinical trials is recognized by clinicians, whose perception of tolerability-related treatment burden often differs from the patient experience [1,2]. The reporting is further complicated by serious AEs, which may necessitate withdrawal from a study but may not represent a significant perceived burden to the individual patient, such as increased QT intervals (i.e., the time between the start of the Q wave and the end of the T wave in the electrical rhythm of the heart). The need to assess the impact on individual patients is supported by a review of AE data collected as outcome measures in studies of antipsychotic drugs, which concluded that: ‘Patients’ subjective experience of medication should be given more consideration’ [3].

Another limitation of current population-level AE reporting is that it is not comprehensive or consistent across studies. Specifically, full listings of all AEs reported during a study are rarely provided, and frequency cutoffs for reporting (e.g., occurring in more than one patient or in ≥ 5% of patients) vary from publication to publication. Clinical study discontinuation because of an AE has been used as a proxy for tolerability in most meta-analyses, but this approach also only captures partial information about patients’ treatment burden, and assumptions about the reasons for discontinuation may underestimate discontinuations resulting from poor tolerability [4]. Moreover, discontinuation may have multiple contributory causes, making the assessment of the primary reason difficult. Alternate assessments, such as global relative benefit–risk ratios [5], provide more detailed information than discontinuation data alone, but are not sufficiently comprehensive or readily translatable to other medications and do not allow simple direct or indirect comparison of treatment burden and tolerability across multiple medications. For example, in the treatment of patients with schizophrenia, poor treatment tolerability can impose a significant patient burden; however, based on the most commonly reported AEs associated with different schizophrenia medications, no single medication has been determined to have a ‘better’ AE profile when the full spectrum of frequency, severity, and seriousness are all taken into account [6]. Characterization of the burden and tolerability of different schizophrenia medications from the patient perspective is even more limited.

This limitation is exemplified by the following description of AEs from a clinical trial of a medication formerly in development for the treatment of patients with schizophrenia:

Discontinuation due to adverse events (AEs) was comparable across all treatment groups. Bifeprunox 30 mg was associated with decreased levels of prolactin at endpoint compared with placebo, while risperidone treatment increased prolactin levels. Bifeprunox and placebo had similar incidence (30 mg bifeprunox: 6%; 40 mg bifeprunox: 8%; placebo: 5%) of EPS while the risperidone group had a greater incidence (14%) of EPS versus placebo … The most common AEs for patients on bifeprunox (incidence >5% and twice for placebo) included: nausea, vomiting, constipation, dyspepsia, diarrhea, dizziness, and decreased appetite. [7]

Although this type of data is helpful because it identifies the incidences and types of AEs, it does not report the likelihood of the individual patient experiencing any AEs, nor does it convey important information about the severity, duration, or burden of the AEs.

The most commonly described categories of antipsychotic AEs include extrapyramidal symptoms (EPS), sedation, weight gain or obesity, metabolic syndrome, diabetes, dyslipidemia, cardiovascular effects, increased prolactin, and sexual dysfunction [8–10]. In addition, patients may experience several other less commonly reported AEs. Indeed, a systematic review of 53 studies concluded that: ‘Antipsychotic adverse effects are diverse and frequently experienced, but are not often systematically assessed’ [9]. The tolerability-related burden associated with these AEs may lead to poor adherence or earlier treatment discontinuation and, as a result, worse functional outcomes [2,11,12]. Several observer-reported and self-reported AE assessment scales for antipsychotic medications are available [2,13–17]. Self-reported scales include the Udvalg for Kliniske Undersøgelser (UKU) Side-Effect Rating Scale, the Liverpool University Neuroleptic Side-Effect Rating Scale (LUNSERS), and the Glasgow Antipsychotic Side-Effect Scale (GASS). The 48-item UKU is one of the most complete scales developed to date [18]. However, the UKU can take as long as 60 minutes to complete and is used largely as a research instrument; it has not been included in pivotal studies for approved antipsychotics. Further, the UKU was developed only for first-generation antipsychotics. The 41-item LUNSERS, developed in 1995 [19] and largely derived from the UKU [16], only measures certain categories of AEs and lacks comprehensiveness. As such, the LUNSERS cannot be readily used to compare the tolerability profiles of antipsychotics and has not been included in many pivotal studies. The GASS resolves some ambiguity of phrasing in the LUNSERS [2] and enables patients to report whether an AE is ‘distressing’ in addition to rating its severity [17]; however, like the LUNSERS, the GASS contains only a subset of the AEs that can occur with antipsychotics and therefore has limited utility. Another approach to estimating AEs is to consider the subjective nature of the AE. Many side-effects associated with antipsychotic drugs can be considered inherently subjective. The Subjective Side-Effect Scale (SSES) is a 22-item battery that provides patient ratings of distress in relation to 11 possible side-effects of antipsychotic medications [20]. This assessment was included in a large cohort study that showed that side-effects, whether subjective or objective, may need to be considered individually in relation to their impact on quality of life [21]. A shortcoming of many self-reported antipsychotic AE questionnaires is that differences among these tools (e.g., individual items included, overall scope) impede direct comparison of their results. For example, in a comparison of four self-rating scales completed by 320 patients with schizophrenia, results did not strongly correlate across questionnaires, at least in part because of the items measured [16].

The objective of the current analysis was to develop a tolerability index for schizophrenia treatments that combines the frequency, severity, duration, and patient-perceived importance attributed to AEs into a single score; includes the patient perspective; can be computed on any dataset that includes patient-level AE data; and provides a scoring system that allows characterization and direct comparison of the overall tolerability of medications used for treating patients with schizophrenia. This article describes initial tolerability index algorithm definition efforts and proof-of-concept testing.

## Methods

Longitudinal AE data, reported from a randomized, placebo-controlled clinical trial from a discontinued antipsychotic medication development program, were used in index development. The 72-day trial evaluated two doses of the discontinued medication bifeprunox (30 mg, *n* = 148; 40 mg, *n* = 148), an active reference medication (risperidone 6 mg, *n* = 154), and placebo (*n* = 149). Placebo was used as reference. By the nature of placebo, it was expected to have a lower burden score compared with active treatments. See Supplemental File 1 for details of calculations.

An expert group, composed of three psychiatrists, a general practitioner, and two experts in data analytics, was assembled to review the study methodology and provide insights on the results. A total of three face-to-face meetings were organized. The first meeting was to define and review the approach. Interim results were reviewed in the second meeting, and consensus on the final algorithm recommendation was reached in the final meeting.

### Unadjusted burden

The unadjusted AE burden represents the number of AEs reported for a given patient (i.e., AE absolute number), and the unadjusted burden score for each treatment arm is equivalent to the mean number of AEs experienced by patients in that arm. If a patient experienced multiple occurrences of the same AE, each event is counted independently.

### Adjusted burden

#### Burden adjusted by AE severity

Severity was expressed on a linear scale, with each AE rating coded from the original clinical trial using the following multipliers: 1 = mild, 2 = moderate or 3 = severe (e.g., three mild AEs impose a burden similar to one severe AE).

#### Burden adjusted by AE severity-adjusted duration

Duration in days was also established from the trial data. For AEs without a recorded end date (e.g., ongoing at study completion), the duration was estimated as ([Date of study end – Date of AE start] + 1). For the purposes of algorithm development, a 72-day study duration was selected, as this is a common duration for studies of antipsychotic drugs. The formulae used will be investigated for adaptation to other study durations.

Duration and severity were combined with a mathematical formula that takes into account the differences in scales so that short duration severe AEs were not masked by medium to long duration mild AEs.

#### Burden adjusted by patient-experienced importance

The patient-experienced importance of the AEs was incorporated into the calculations to derive an adjusted AE burden. For the purposes of initial algorithm development, it was necessary to estimate the importance of the AE to the patient on a scale of 0 (least important) to 1.0 (most important). As the objective of the study was to test the feasibility of the development of a tolerability index, it was not feasible to collect burden directly from the patients; an independent psychiatrist served as a proxy for the patient-rated AE burden during this stage of the development. For the purpose of this proof of concept of patient burden, the assignment of the importance of the AE was performed by a single psychiatrist who was not otherwise involved in this project. The psychiatrist’s ratings were performed *post hoc* using AE data from a randomized, placebo-controlled clinical trial [7]. For AEs not scored by the psychiatrist, importance factors were imputed by calculating the average factor for other AEs in the same system organ class. A total of 604 different AEs rated by the psychiatrist were reported in the study. To estimate their weight, 462 were estimated by system organ class average and 142 were estimated by overall importance factor average because their system organ class did not correspond to any of the system organ class AEs scored by the expert. Higher importance factors indicate a greater perceived AE impact. AEs leading to discontinuation were assigned an importance factor of 1.0 for that patient. See Supplementary Table 1 for assigned importance factors for AEs.

**Table 1.** Mean treatment burden scores (unadjusted and adjusted).

Score	Bifeprunox 30 mg (*n* = 148)	Bifeprunox 40 mg (*n* = 148)	Risperidone 6 mg (*n* = 154)	Placebo (*n* = 149)
Unadjusted				
Mean	2.91	2.96	3.05	2.01
Median (range)	2.00 (0–24.00)	2.00 (0–14.00)	2.00 (0–18.00)	1.00 (0–12.00)
Adjusted by patient-experienced impact				
Mean	1.85	1.92	2.01	1.31
Median (range)	1.12 (0–15.09)	1.20 (0–9.29)	1.50 (0–11.75)	0.90 (0–7.73)
Adjusted by severity				
Mean	4.04	4.26	4.16	2.99
Median (range)	2.00 (0–41.00)	3.00 (0–22.00)	3.00 (0–33.00)	2.00 (0–21.00)
Adjusted by severity-adjusted duration				
Mean	8.43	8.74	8.98	6.16
Median (range)	6.02 (0–45.44)	6.11 (0–40.64)	6.61 (0–40.36)	3.67 (0–30.59)
Adjusted by patient-experienced impact and severity-adjusted duration				
Mean	7.64	8.40	8.14	6.15
Median (range)	4.30 (0–45.63)	5.42 (0–40.27)	6.34 (0–48.90)	4.10 (0–49.43)

### Discriminatory capability of burden scores

The discriminatory capability of adjusted versus unadjusted burden scores was evaluated using AE-related discontinuation data from the selected clinical trial. Mean burden scores (unadjusted, adjusted by experienced impact, adjusted by severity, adjusted by severity-adjusted duration, and adjusted by both experienced impact and severity-adjusted duration) were calculated for patients who discontinued because of an AE (*n* = 73; 14%) or who did not discontinue because of an AE (*n* = 526; 86%) to validate the analysis of AE burden and the determination of burden scores. The discriminatory capability of unadjusted and adjusted burden scores was analyzed using odds ratios calculated by logistic regression for patients who did or did not discontinue treatment. Univariate analyses were conducted where exploratory variables were unadjusted index, index adjusted on severity, index adjusted on weight, and treatment arms. Odds ratios (95% CIs) and *P* values were computed.

### Tolerability index

To facilitate interpretation and comparison across different studies and treatments, a tolerability index algorithm is proposed that normalizes treatment burden scores on a 0 to 100 scale. The tolerability index score for each patient was calculated using burden scores adjusted by the patient-experienced importance factor and severity-adjusted duration of AEs using the following equation:

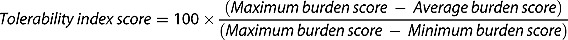

The maximum and minimum burden scores represent the range of computed burden scores for all patients in the study and the average burden score is specific to a given patient. Using this formula, the tolerability index score (possible score range, 0–100) is based on, and is inversely proportional to, the treatment burden experienced by a patient during the study. Tolerability index scores for each treatment arm were computed by averaging the tolerability index scores for all patients within that arm. Higher tolerability scores indicate less treatment burden and greater tolerability.

## Results

### Unadjusted burden

**Figure 1.**
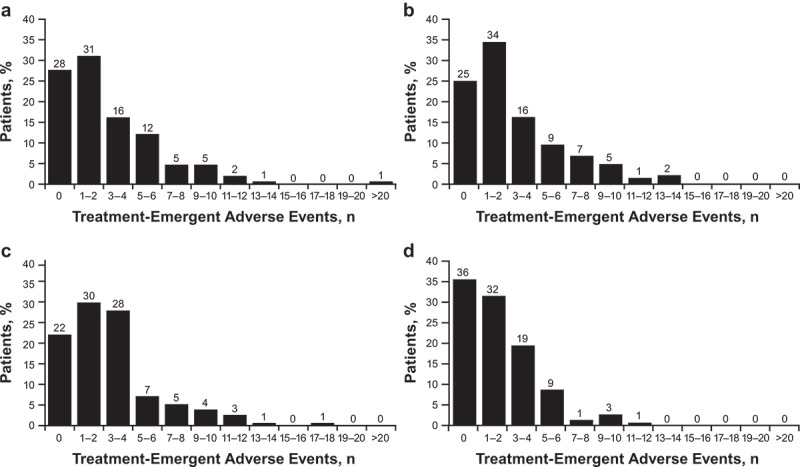
Frequency of treatment-emergent adverse events. (a) Bifeprunox 30 mg (*n* = 148); (b) bifeprunox 40 mg (*n* = 148); (c) risperidone 6 mg (*n* = 154); (d) placebo (*n* = 149).

The unadjusted burden (i.e., total number of AEs reported) for each patient across treatment arms ranged from 0 to 24. The relative frequency of patients who experienced a given number of AEs from 0 to the maximum number of AEs is presented in Figure 1. The maximum frequency of 24 AEs was reported for a patient in the bifeprunox 30 mg treatment arm. The mean unadjusted treatment burden scores (reflecting the mean number of AEs experienced by patients in that arm) for bifeprunox 30 mg, bifeprunox 40 mg, risperidone 6 mg, and placebo were 2.91 (range, 0–24), 2.96 (range, 0–14), 3.05 (range, 0–18), and 2.01 (range, 0–12), respectively (Table 1). Using the unadjusted burden scores, placebo was ranked as the least burdensome and risperidone 6 mg was ranked as the most burdensome.

### Adjusted burden

#### Burden adjusted by AE severity

Individual burden scores adjusted by AE severity are summarized in Table 2. These data reflect the burden associated with the frequency of multiple AEs of varying severity. Mean treatment burden scores adjusted by severity for bifeprunox 30 mg, bifeprunox 40 mg, risperidone 6 mg, and placebo were 4.04, 4.26, 4.16, and 2.99, respectively (Tables 1 and 2). Placebo was ranked as the least burdensome and bifeprunox 40 mg was ranked as the most burdensome.

**Table 2.** Burden score adjusted by AE severity.

AEs,	Bifeprunox 30 mg		Bifeprunox 40 mg		Risperidone 6 mg		Placebo	
***n* and severit**y^a^	AEs, *n*	**Burden**^b^	AEs, *n*	**Burden**^b^	AEs, *n*	Burden^b^	AEs, *n*	**Burden**^b^
1 AE								
Mild	22	22	17	17	20	20	10	10
Moderate	6	12	8	16	1	2	9	18
Severe	3	9	3	9	3	9	3	9
2 AEs								
Mild	17	17	27	27	26	26	28	28
Moderate	12	24	14	28	15	30	20	40
Severe	1	3	5	15	3	9	2	6
10 AEs								
Mild	39	39	21	21	25	25	7	7
Moderate	1	2	9	18	5	10	11	22
Severe	0	–	0	–	0	–	2	6
> 20 AEs								
Mild	8	8	0	–	0	−	0	−
Moderate	15	30	0	–	0	−	0	−
Severe	1	3	0	–	0	−	0	−
Total burden		598		631		641		445
Mean burden score		4.04		4.26		4.16		2.99

Representative data are presented for patients experiencing 1, 2, 10, or > 20 AEs (e.g., of the 31 patients who received bifeprunox 30 mg and experienced only one AE, 22 experienced a mild AE, six experienced a moderate AE, and three experienced a severe AE).

AE: treatment-emergent adverse event.

^a^AE severity was scored as mild = 1, moderate = 2, and severe = 3.

^b^Burden scores were calculated by multiplying the AEs for each severity ranking by the score assigned to that ranking and adding the values to determine the total burden.

#### Burden adjusted by AE severity-adjusted duration

Mean treatment burden scores adjusted by AE severity-adjusted duration for bifeprunox 30 mg, bifeprunox 40 mg, risperidone 6 mg, and placebo were 8.43, 8.74, 8.98, and 6.16, respectively (Table 1). Placebo was ranked as the least burdensome and risperidone 6 mg was ranked as the most burdensome.

#### Burden adjusted by patient-experienced importance

Independent expert-assigned importance factors (an estimate of patient-experienced impact based on clinical importance) for the most common AEs ranged from 0.30 for increased appetite and peripheral edema to 0.90 for aggravated psychosis, psychotic disorder, tardive dyskinesia, and diabetes mellitus (see Supplementary Table 1 for assigned importance factors for AEs). Mean treatment burden scores adjusted by patient-experienced importance for bifeprunox 30 mg, bifeprunox 40 mg, risperidone 6 mg, and placebo were 1.85, 1.92, 2.01, and 1.31, respectively (Table 1). Placebo was ranked as the least burdensome and risperidone 6 mg was ranked as the most burdensome.

Mean treatment burden scores adjusted by patient-experienced importance and severity-adjusted duration of AEs for bifeprunox 30 mg, bifeprunox 40 mg, risperidone 6 mg, and placebo were 7.64, 8.40, 8.14, and 6.15, respectively (Table 1). Placebo was ranked as the least burdensome and bifeprunox 40 mg was ranked as the most burdensome.

### Discriminatory capability of burden scores

The discriminatory capability of unadjusted and adjusted burden scores was evaluated using data for subgroups of patients who discontinued or did not discontinue treatment because of AEs. Mean burden scores were higher for patients who discontinued than for those who did not, regardless of burden score adjustment (Table 3). The ratio of scores for patients who did versus did not discontinue because of AEs was greater for adjusted burden scores than for unadjusted burden scores. Percentages of patients who discontinued because of AEs were similar across treatment arms; however, burden scores were significantly higher in the discontinuation subgroup and could be used to discriminate between patients who discontinued because of AEs compared with those who did not (Table 4).

**Table 3.** Burden scores based on AE-related treatment discontinuation.

Discontinued Due to AE?	Burden score	Sum	Mean ± SD	Median	Range	**Ratio**^a^
No (*n* = 526)	Unadjusted	1331	2.53 ± 2.97	2	0–18	1.66
	Adjusted by patient-experienced impact	857.85	1.63 ± 1.93	1.03	0–11.75	1.98
	Adjusted by severity	1796	3.41 ± 4.21	2	0–33	2.08
	Adjusted by severity-adjusted duration	3921.37	7.46 ± 8.49	4.14	0–45.45	1.69
	Adjusted by patient-experienced impact and severity-adjusted duration	3447.63	6.55 ± 7.92	3.71	0–48.90	2.29
						
Yes (*n* = 73)	Unadjusted	306	4.19 ± 3.77	3	0–24	
	Adjusted by patient-experienced impact	235.3	3.22 ± 2.56	2.43	0–15.88	
	Adjusted by severity	519	7.11 ± 6.03	5	0–41	
	Adjusted by severity-adjusted duration	921.33	12.62 ± 9.50	9.52	0–39.62	
	Adjusted by patient-experienced impact and severity-adjusted duration	1095.21	15.00 ± 10.26	11.07	0–49.93	

SD: standard deviation; AE: treatment-emergent adverse event.

^a^The ratio reflects the mean tolerability index score for patients who discontinued because of an AE divided by mean tolerability burden index score for patients who did not. A higher ratio indicates a higher discriminatory property of the methods used to calculate the burden score.

**Table 4.** Validation of burden scores: discontinuations due to AEs.

	Discontinued due to AEs?			
	No (*n* = 526)	Yes (*n* = 73)	**Odds ratio (95% CI)**^c^	*P* value
**Burden score, mean ± SD**^a^				
Unadjusted	2.53 ± 2.97	4.19 ± 3.77	1.15 (1.07–1.22)	<0.0001
Adjusted by severity	3.41 ± 4.21	7.11 ± 6.03	1.14 (1.09–1.19)	<0.0001
Adjusted by patient-experienced impact^b^	1.63 ± 1.93	3.22 ± 2.56	1.33 (1.20–1.47)	<0.0001
**Treatment, *n* (%)**				0.7328
Bifeprunox 30 mg	132 (25.10)	16 (21.92)	0.70 (0.35–1.39)	0.5692
Bifeprunox 40 mg	131 (24.90)	17 (23.29)	0.75 (0.38–1.48)	0.7860
Risperidone 6 mg	136 (25.86)	18 (24.66)	0.76 (0.39–1.49)	0.8525
Placebo	127 (24.14)	22 (30.14)	1	

SD: standard deviation; AE: treatment-emergent adverse event.

^a^Data are presented for 1-unit increments.

^b^A weight of 1 was applied for AEs leading to discontinuation.

^c^Odds ratios were calculated using a logistic regression.

### Tolerability index score

**Figure 2.**
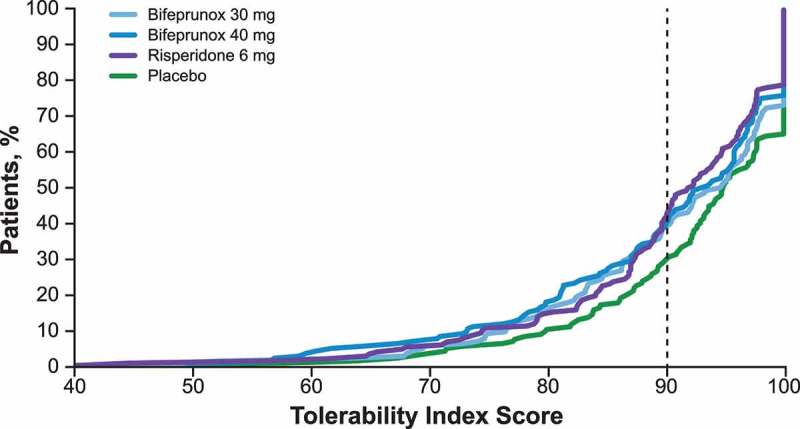
Cumulative distribution of tolerability index scores by study arm.

The tolerability index algorithm was proposed to allow standardized comparison of tolerability across studies and treatments. Tolerability index scores were derived from burden scores adjusted by experienced impact and severity-adjusted duration. The mean treatment tolerability index scores for bifeprunox 30 mg, bifeprunox 40 mg, risperidone 6 mg, and placebo were 90.44, 89.50, 89.82, and 92.31, respectively, with higher tolerability index scores (possible range, 0–100) indicating better tolerability. Tolerability scores were generally similar across treatment arms. The distribution of individual patients’ tolerability index scores across treatment arms indicated population-level differences among treatments. Compared with placebo, fewer patients receiving bifeprunox or risperidone had tolerability index scores > 90 (Figure 2). Specifically, tolerability index scores > 90 were reported for > 75% of patients receiving placebo compared with approximately 65%, 60%, and 60% of patients receiving bifeprunox 30 mg, bifeprunox 40 mg, and risperidone 6 mg, respectively.

## Discussion

Adverse event reporting is a standard requirement in regulatory clinical trials and is consistently reported in most trials. Although summaries of the frequency of commonly reported AEs provide useful information about treatment safety, they provide somewhat limited information about treatment tolerability from the patient perspective. Standard AE reporting methods reflect the patient perspective as filtered through or modified by the physician’s perspective. Because tolerability can be an important factor in treatment adherence and success, there is a need for tools that account for patient perspectives in the assessment of treatment tolerability. The goal of these analyses was to develop a tolerability index that accounts for the burden imposed by the drug on the patient. Treatment-experienced impact, severity, and duration of AEs, in addition to frequency, were assessed to provide a patient-level view of treatment tolerability. Proof-of-concept testing was performed using AEs reported from a randomized, placebo- and active-controlled clinical trial of an antipsychotic medication that was in development for schizophrenia; for these initial development efforts, patient perspectives were estimated by an experienced physician. As this model is refined and tested using additional clinical study data, it will be important to incorporate information obtained directly from patients. Burden score adjustments were calculated and their discriminatory capability was evaluated by comparing subgroups of patients who discontinued versus did not discontinue because of AEs; next, tolerability index scores were calculated based on adjusted burden to normalize scores on a scale of 0 to 100. A major assumption of this model is that AEs leading to discontinuation were assigned an importance factor of 1.0 for the patient. Future studies will include sensitivity analyses to test this assumption (e.g., using importance factor values for AEs leading to discontinuation other than 1.0). As expected, burden and tolerability index scores were most favorable for placebo compared with active treatments and worsened with increasing drug dosage. This approach was taken to avoid overestimating the burden for mild but long-lasting AEs or underestimating the burden for severe AEs of a short duration.

Based on the results of these analyses and factors that may influence the practicality of implementing the tolerability index in future studies, the authors and members of an advisory committee determined that calculation of the tolerability index using treatment burden adjusted for the combination of AE frequency and patient-experienced importance would provide an optimal combination of precision and simplicity. This recommendation is based on several factors. Frequency and patient-experienced importance are intuitively easy for clinicians to understand and, although duration is an important factor in treatment tolerability, it is less practical as an AE adjustment because the severity-adjusted duration calculations are complex. In practice, duration of the most severe AEs may be difficult to estimate because these AEs would lead to discontinuation and would therefore require several assumptions. Further, the advisory committee proposed that the patient-experienced importance of AEs as reported directly by patients would intrinsically incorporate information on the severity and duration of the AEs experienced to account for both the quantitative and qualitative aspects of AEs (similar to the quality-adjusted life-year approach). Therefore, AE duration would be captured indirectly in patient-reported AE importance rather than as an individual, clinician-reported variable. Finally, the results of these analyses indicated that the precision of the tolerability index was comparable when AEs are adjusted by frequency and patient-experienced impact compared with adjustment by more variables (i.e., frequency and patient-experienced impact in addition to severity and duration).

For the purposes of tolerability index algorithm development and proof-of-concept testing, a single psychiatrist assigned values for the patient-experienced importance of AEs. Because these values served as a proxy for patient-provided AE importance values for the experienced impact in the current analyses, the patient perspective was not fully captured in the current implementation. Future testing of the tolerability index will use AE importance factors provided directly by patients. The interaction effect of multiple types of AEs of varying levels of patient-experienced impact was not addressed in these proof-of-concept calculations designed to assess the feasibility of the index. More sophisticated methods, such as the discrete choice experiment (DCE) method, will be used to apply actual patient perspectives to AE importance factors. The value of DCEs has been demonstrated in previous studies of patients with asthma or psoriasis [22,23]. In these studies, the DCE approach was applied to patient-reported preference data to identify patient-perceived benefits and risks or tradeoffs that influenced their choice of treatment [22,23]. The DCE approach will be used to assign factors corresponding to the patient-experienced impact of AEs reported by patients in interviews or focus groups to provide a sophisticated measure of tolerability-related treatment burden from the patient perspective. The need for incorporation of patient perspectives in tolerability assessments was recently emphasized by Dr Richard Pazdur, director of the US Food and Drug Administration Office of Hematology and Oncology Products, who stated: ‘Instead of having physicians assess drug toxicities, which they do a terrible job of, we’re going to start asking patients to assess them. It’s a huge issue’ [24]. The disparity between patient-reported and clinician-reported symptoms can negatively impact patient care [25], with one study indicating that healthcare providers underestimated the percentages of moderate or severe symptoms relative to patients’ reports of symptom intensity [26].

Because some AEs, such as laboratory values (e.g., liver enzymes), will by their nature require physician reporting rather than patient reporting, an imputation method will be defined to attribute importance factors to the experienced impact of these AEs. Together, these approaches will provide a comprehensive approach to assessing treatment tolerability that captures relevant AEs and patient-level perspectives. External validation of the tolerability index will be performed using a scale such as the UKU [18] or LUNSERS [19] self-assessment tool. A benefit of the tolerability index approach is that it can be adapted to other treatments or conditions such as major depressive disorder. AE weighting based on patient-experienced impact must be adjusted to account for the side-effect profiles and patient-reported tolerability of the medications and disease states assessed; however, the index can be implemented retrospectively and without any additional data collection. Further, AE data collected during a clinical trial were used in development of the tolerability index algorithm; the index can also be used for datasets, including only treatment-related AEs (i.e., adverse drug reactions), to provide an additional measure of treatment tolerability.

The importance of concomitant versus sequential AEs was not tested (i.e., experience of a first AE followed by a second AE, vs the experience of two AEs at the same time). Although it is possible that concomitant rather than sequential AEs could affect the burden, it was too complex to model in this scenario. Another possible limitation is that for some patients (and healthcare providers), the experience of an AE may be seen as evidence of the drug effect (i.e., the drug is working). The influence of this factor on the burden would be very complex to model and was not included to allow the index to remain as straightforward as possible.

Use of pivotal trial data with a fixed dosing study design, while providing necessary detail for this index, does introduce the limitation that the efficacy to tolerability ratio may not be fully optimized because the dose cannot be adjusted for individual patients. Derivation of the actual patient-level impact (i.e., importance factor) of each type of AE will require additional experimental work to incorporate the patient perspective. It is expected that patient-experienced importance factors derived in this study will demonstrate a greater range than has been shown in the current analysis.

## Conclusions

The proposed tolerability index incorporates a more patient-centric method of AE reporting that reflects the fact that most patients experience multiple different AEs. Although further algorithm validation and elicitation of patient-reported importance factors for the perceived impact of the AEs are ongoing, these initial proof-of-concept analyses suggest that standardized scoring of treatment burden and tolerability is both possible and clinically relevant. The tolerability index approach may enable quantitative comparison of tolerability profiles across multiple medications used in treating patients with schizophrenia. The approach described here has the potential to be used on any dataset without the need for additional data collection, and can be adapted for use in evaluating the tolerability of medications used to treat a variety of conditions.
